# Serological differentiation of antibodies against *Rickettsia helvetica*, *R. raoultii*, *R. slovaca, R. monacensis* and *R. felis* in dogs from Germany by a micro-immunofluorescent antibody test

**DOI:** 10.1186/s13071-015-0745-1

**Published:** 2015-03-23

**Authors:** Miriam Wächter, Silke Wölfel, Martin Pfeffer, Gerhard Dobler, Barbara Kohn, Andreas Moritz, Stefan Pachnicke, Cornelia Silaghi

**Affiliations:** Comparative Tropical Medicine and Parasitology, Ludwig-Maximilians-University Munich, Leopoldstrasse 5, 80802 Munich, Germany; Department of Virology and Rickettsiology, Bundeswehr Institute of Microbiology, Neuherbergstrasse 11, 80937 Munich, Germany; DZIF German Centre for Infection Research–Ludwig-Maximilians-University Munich, Geschwister-Scholl-Platz 1, 80539 Munich, Germany; Institute of Animal Hygiene and Veterinary Public Health, University of Leipzig, An den Tierkliniken 1, 04103 Leipzig, Germany; Small Animal Clinic, Faculty of Veterinary Medicine, Freie Universität Berlin, Oertzenweg 19b, 14163 Berlin, Germany; Department of Veterinary Clinical Sciences, Clinical Pathology and Clinical Pathophysiology, Justus-Liebig-University, Frankfurterstrasse 126, 35392 Gießen, Germany; Bayer Vital GmbH, 51368 Leverkusen, Germany; Current affiliation: National Centre for Vector Entomology, Institute of Parasitology, University of Zurich, Winterthurerstrasse 266a, 8057 Zurich, Switzerland

**Keywords:** *Rickettsia helvetica*, *R. raoultii*, *R. slovaca*, *R. monacensis*, *R. felis*, Seroprevalence, Differentiation, Dogs, Ticks

## Abstract

**Background:**

Spotted Fever Group (SFG) Rickettsiae can cause febrile diseases with or without rash in humans worldwide. In Germany only limited data are available about their medical significance. Serological screening tests for antibodies against rickettsiae usually only distinguish between SFG and Typhus Group (TG) Rickettsiae due to the strong cross reactivities within the groups. Seroprevalence rates in dogs, as possible sentinels for tick-borne diseases, could serve as an indicator for the distribution of different *Rickettsia* species.

**Methods:**

In this study, a micro-immunofluorescence assay (micro-IFA) was established for detection and differentiation of antibodies against five *Rickettsia* species in dogs (*R. helvetica*, *R. raoultii*, *R. slovaca*, *R. monacensis* and *R. felis*). Dogs that never left Germany (n = 605) previously investigated with an SFG-ELISA were included in this study and screened at a 1:128 dilution. Endpoint titres of fifty randomly selected seropositive samples of each of the five investigated regions in Germany were determined in order to allow a differentiation of the causative *Rickettsia* species. Sensitivity and specificity of the micro-IFA were compared with ELISA results of the previous study.

**Results:**

A total of 93.9% of the dogs were positive for antibodies of the SFG Rickettsiae at the screening titer of 1:128. Differentiation of SFG Rickettsiae with the micro-IFA was possible in 70.4%, but in 29.6% of the cases the detected antibodies were not differentiable. Considering a clear differentiation by a twofold titre difference between observed reactions, the seroprevalence rates were 66.0% for *R. helvetica,* 2.8% for *R. raoultii,* 1.6% for *R. slovaca*, but no serological reaction could be clearly attributed to *R. monacensis* or *R. felis*. No statistically significant regional differences were found for *R. helvetica*, *R. slovaca* and *R. raoultii* comparing the five regions of Germany. Comparison of micro-IFA with ELISA revealed a sensitivity of 82.0% and a specificity of 83.8% for the *Rickettsia* SFG ELISA.

**Conclusions:**

The micro-IFA is a useful serological tool to differentiate antibodies against different *Rickettsia* species in dogs. Seroprevalence rates in dogs correspond to the prevalence rates and distribution of *Rickettsia*-carrying tick species.

## Background

Rickettsiae are short rod-shaped gram-negative, obligate intracellular bacteria [1]. They are, regarding the clinical and serological perspective, divided into two major groups: the Typhus Group (TG) and the Spotted Fever Group (SFG). The TG includes two members: *R. typhi* and *R. prowazekii*, the causative agents of murine typhus and epidemic typhus, respectively. The heterogeneous SFG contains >20 Rickettsiae, mainly transmitted by ticks, except for *R. felis* which is transmitted by fleas and *R. akari* which is transmitted by mites [2-4]. Recent genomic analyses indicate the divison into four groups: the TG, the SFG, the ancestral group (AG) and the transitional group (TRG), which includes *R. felis* [5]. Rickettsiae of the SFG are able to cause mild to severe rickettsioses in humans [6]. Rickettsioses are considered emerging infectious diseases worldwide [1,7]. To evaluate the epidemiological situation in different countries it is necessary to examine vectors and reservoir hosts for the occurrence of different rickettsial species. Various molecular and serological methods have been described for the detection and differentiation of *Rickettsia* species. Real-time polymerase chain reaction (PCR) is frequently applied to detect *Rickettsia* in biopsies, blood and arthropods [7]. Several conventional PCRs targeting different genomic regions for subsequent sequencing and phylogenetic analysis of rickettsiae have been published in the last decades [4,8,9]. Species-specific real-time PCRs are only available for some rickettsial species (e.g. *R. conorii*, *R. rickettsii*) [10,11]. Due to the strong serological cross-reactions among SFG Rickettsiae, the availability of serological tools for differentiation is limited, so far. Commercially available IFA and ELISA tests are only suitable for the discrimination between SFG- and TG-Rickettsiae [1]. Brouqui et al. [12] described a micro-immunofluorescence method which allows the investigation of up to nine different rickettsial antigens in one spot as a suitable method to differentiate the causative *Rickettsia* species by determination of endpoint titres. Absorption western blotting can also be used to identify rickettsial species [13]. The rickettsial IFA adapted to the micro-method format (micro-IFA) is the test of choice for the serodiagnosis of rickettsial disease in human medicine [1]. In Germany, six species of the SFG Rickettsiae have been detected in ticks by molecular methods (Table 1). All of them have been described to cause diseases in humans. *R. helvetica* is generally associated with uneruptive fever, but cases with more severe clinical signs such as endocarditis or meningitis have been reported [14-16]. *R. monacensis*, *R. massiliae* and *R. felis* can cause classical spotted fevers and additional constitutional symptoms like fatigue, headache and myalgia [7,17-23]. Nevertheless the pathogenicity of *R. felis* is still discussed controversially [24]. *R. raoultii* and *R. slovaca* cause TIBOLA (tick-borne lymphadenopathy) or DEBONEL (*Dermacentor*-borne necrosis erythema lymphadenopathy) with a typical clinical syndrome including an eschar at the site of the tick bite and lymphadenopathy [25-27]. Data concerning clinical human cases in Germany are rare. In the recent years only a few cases have been published, among them cases of *R. slovaca* infection in 2009 and one in 2010 [27,28] and a case of *R. felis* infection in 2000 [29]. So far no clinical cases caused by *R. monacensis*, *R. helvetica*, *R. massiliae* or *R. raoultii* were described in Germany. In 2008, 9.1% of 256 examined hunters in Germany had antibodies against the SFG Rickettsiae in an IFA [30]. In 2012, an average of 27.7% of forestry workers had antibodies against the SFG Rickettsiae in the IFA with seroprevalences up to 55% in particular geographical regions (Wölfel et al., Seroprevalence of IgG against Rickettsiae of the Spotted Fever Group in Forestry Workers in State Brandenburg, Eastern Germany, unpublished). In 2014, we detected antibodies against the SFG Rickettsiae in 77.9% of 605 dogs examined with ELISA (Wächter et al., Seroprevalence of Spotted Fever Group Rickettsiae in dogs in Germany, in press). Until now no clinical cases in dogs involving the rickettsial species *R. helvetica*, *R. raoultii*, *R. slovaca*, *R. massiliae*, *R. felis* or *R. monacensis* have been reported. However, *R. rickettsii* in the USA and *R. conorii* in southern Europe can cause symptomatic diseases of variable severity in dogs [31,32]. *Rickettsia rickettsii* causes a severe vasculitis leading to symptoms like lethargy, anemia and neurologic symptoms [31]. *Rickettsia conorii*, causing the Mediterranean Spotted Fever in humans, can lead to symptoms like fever, diarrhea, vomiting and petechial rash in dogs [32]. In our previous study statistically significant correlations between age, tick infestation and seropositivity of the dogs were found, but no differentiation of rickettsial species was possible by ELISA (Wächter et al., Seroprevalence of Spotted Fever Group Rickettsiae in dogs in Germany, in press). Therefore, the aims of the study were (i) to establish the micro-IFA as a tool to distinguish antibodies against different rickettsial species in dogs (ii) to differentiate the antibody responses in dogs previously found positive by ELISA and (iii) to compare the micro-IFA to a commercial ELISA with regard to sensitivity and specificity.

**Table 1:** **Prevalence rates of Spotted Fever Group Rickettsiae in ticks in Germany, according to the geographic region**

***Rickettsia*** **species**	**Tick species**	**% pos**	**Number of ticks tested**	**Region**	**Reference**
*R. helvetica*	*Ixodes ricinus*	5.6-13.3%	1187	BW	[33]
		12.0%	2141	BY	[34]
		4.8%	2861	BY	[35]
		6.0%	4459	BY	[36]
		14.2%	127	BE	[37]
		3.5-6.2%	3591	south of Germany	[7]
		32.9%	1089	NI	[38]
		13.4-17.4%	1702	BY/ SL/ SN	[39]
		8.5%	2186	BY	[40]
		52.5%	1400	Hamburg	[41]
		26.2%	2100	Hannover	[42]
*R. raoultii*	*Dermacentor*	30.0%	666	BW	[43]
	spp.	56.7%	1359	BY/ SL/ SN	[39]
*R. slovaca*	*Dermacentor*	0.8%	666	BY	[43]
	spp.	13.3%	15	BY/ SL/ SN	[39]
*R. monacensis*	*I. ricinus*	0.5%	2861	BY	[35]
		0.6%	3591	BY	[7]
		0.4%	4459	BY	[36]
		0.4%	1089	NI	[38]
		0.2%	2186	BY	[40]
*R. felis*	*I. ricinus*	0.4%	1450	BY/NRW	[7]
*R. massiliae*	*I. ricinus*	1.7%	57	eastern BY	[7]

BE: Berlin; BY: Bavaria; BW: Baden-Wurttemberg; NI: Lower Saxony; NRW: North Rhine-Westphalia; SL: Saarland; SN: Saxony.

## Methods

### Samples

Altogether 605 serum samples of dogs from a previous study (Wächter et al., Seroprevalence of Spotted Fever Group Rickettsiae in dogs in Germany, in press) were tested for antibodies against five SFG Rickettsiae by micro-IFA. These samples had previously been tested for antibodies against SFG Rickettsiae by a commercial ELISA (Canine Spotted Fever Rickettsia EIA IgG Antibody Kit, Fuller Laboratories, Fullerton, California, USA) (Wächter et al., Seroprevalence of Spotted Fever Group Rickettsiae in dogs in Germany, in press). Dogs included in this study were (i) born in Germany, (ii) had never left Germany and (iii) data on sex, age, breed, location, clinical status (healthy or sick), tick infestation and date of blood collection were available.

### Production of micro-IFA-slides

**Figure 1:**
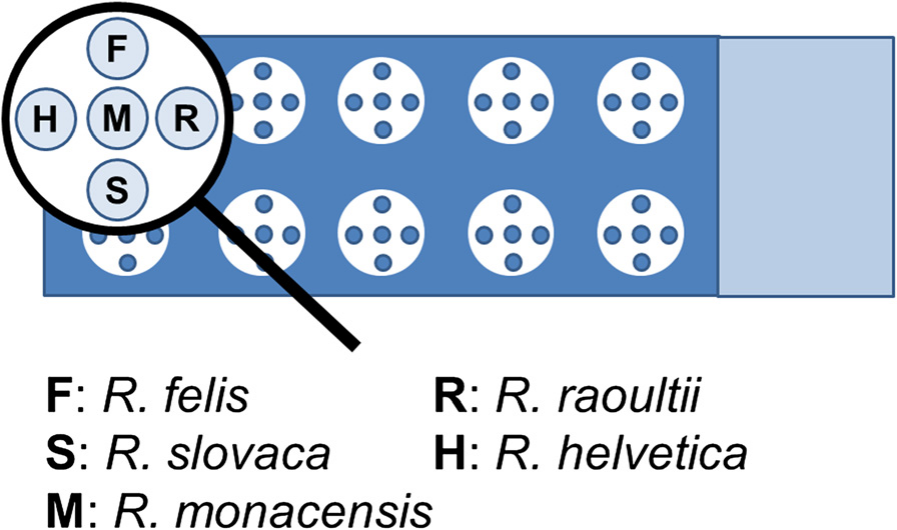
**Arrangement of rickettsial antigen dots per spot on the Micro IFA-Slide.**

*Rickettsia helvetica* strain AS 819, *R. raoultii* strain *Khabarovsk* (kindly provided by Lee Fuller, Fuller Laboratories), *R. slovaca* strain RU 828, *R. monacensis* strain AS 787 and *R. felis* strain ELB (kindly provided by Lee Fuller, Fuller Laboratories) were cultivated in 75 cm^2^ tissue culture flasks containing either Vero cells or Drosophila melanogaster cells (only *R. felis*). Readily infected cell cultures were harvested after 8 to 19 days, depending on the growth characteristics of the respective *Rickettsia* species. Identity and purity of the strains was confirmed by sequencing a part of the ompB gene following a protocol of Roux and Raoult [2]. After inactivation by adding formalin in a final concentration of 1% the Rickettsiae were released from their host cells by needle disruption. The suspension was centrifuged twice at 1,000 g, 4°C for 5 minutes to remove cell debris. The supernatant was collected. Centrifugation at 17,000 g, 4°C for 5 minutes followed twice. Every time the supernatant was discarded and the pellet was resuspended in 1.8 ml phosphate buffered saline (PBS). The final pellet was resuspended in 100 μl PBS. Serial dilutions in PBS (1:2, 1:5, 1:10) of the antigens were applied with a 10 μl piston-operated pipette to empty slides to estimate the density of rickettsial antigen by IFA and Diff-Quick ® (Polysciences, Warrington, USA) staining to equalize the density of all five rickettsial species. *R. felis*, *R. monacensis* and *R. slovaca* were diluted with PBS 1:10, *R. helvetica* and *R. raoultii* were used as undiluted solutions. The different rickettsial antigens were applied to a 10-well microscope slide with a pen-point as shown in Figure 1. The slides were air dried and fixed in methanol-acetone (1:1) for 30 min, air-dried again and stored at 4°C until use.

### Micro-IFA

**Figure 2:**
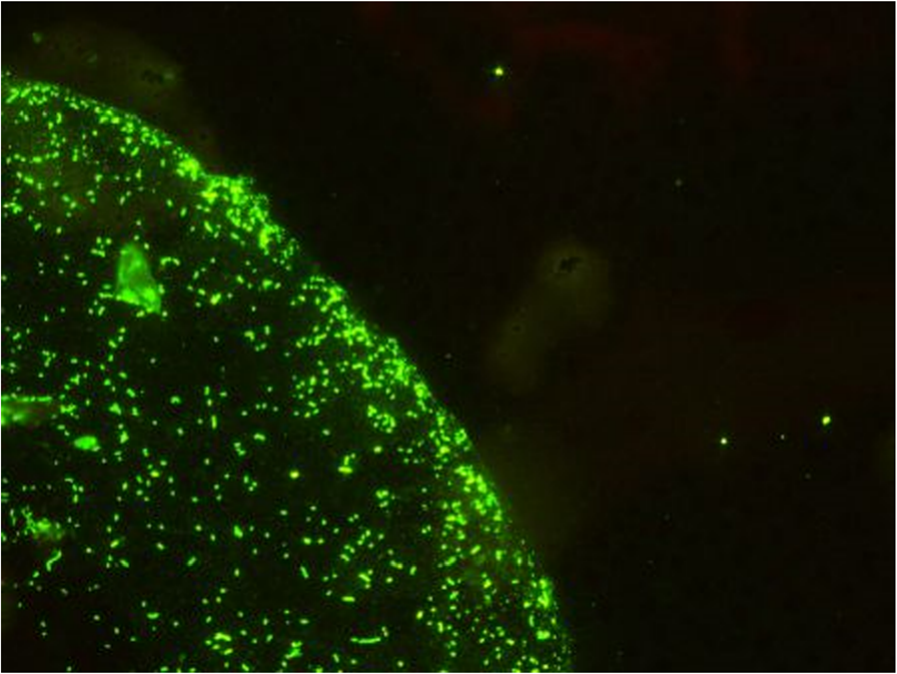
**Positive reaction for antibodies against** ***Rickettsia raoultii*** **antigen on the Micro IFA-Slide (dilution 1:128).**

A twofold serial dilution of the serum samples with PBS was made and the screening dilution of 1:128 was applied to the slide wells and incubated at 37°C for 45 min in a humid chamber. The slide was washed with 0.2% PBS-Tween (Euroimmun, Lübeck, Germany) three times for 5 min. Fluorescein isothiocyanate (FITC)-labelled rabbit anti-dog immunoglobulin G (Sigma-Aldrich, St. Louis, USA) was applied in a 1:20 dilution and incubated at 37°C for 30 min in a humid chamber, washed with 0.2% PBS-Tween three times for 5 min and mounted with fluorescence mounting medium (Dako, Carpinteria, California, USA). Reactions were read with a fluorescence microscope (Leica 5000, Wetzlar, Germany) by two independent investigators. Suitable positive and negative control dog sera that had been identified in a previous study (Wächter et al., Seroprevalence of Spotted Fever Group Rickettsiae in dogs in Germany, in press) were included in each assay. Samples were considered positive for antibodies of the SFG Rickettsiae when any of the five antigens showed distinct fluorescence patterns at the 1:128 dilution (Figure 2). Fifty positive samples of each of the five regions (Figure 1) were chosen with a random generator (source: http://rechneronline.de/zufallszahlen/, 20-05-2014) and subjected to end-point titration. If one rickettsial species showed at least twofold higher antibody titres than the others, it was considered the species responsible for the antibody induction. If more than one species showed fluorescence at the same end point titre the results were considered not distinguishable.

### Comparison ELISA-micro-IFA

The results of the screening dilution (1:128, n = 605) of this study obtained with micro-IFA were compared to results obtained with ELISA from a previous study (Wächter et al., Seroprevalence of Spotted Fever Group Rickettsiae in dogs in Germany, in press).

### Statistical analysis

Statistical analysis was performed with SPSS® version 21.0.1., SPSS Inc., Chicago, IL, USA. Correlations between location and seropositivity were compared by χ^2^-test, for small sample sizes the Fishers exact test was used. Statistical significance was considered at a p-value <0.05. Multiple comparisons were corrected with the Bonferroni adjustment to p < 0.01. Confidence intervals (95% CI) were calculated individually for each proportion with the Clopper and Pearson method. Sensitivity and specificity of the ELISA was calculated as follows: Sensitivity (true positive rate = TPR) = TP (true positive)/ P (positive) and specificity (SPC, true negative rate) = TN (true negative)/ N (negative). The TP and the TN were the sera seropositive and seronegative for antibodies for the SFG tested with the micro-IFA, P and N were the sera tested seropositive and seronegative with the ELISA, respectively.

## Results

**Figure 3:**
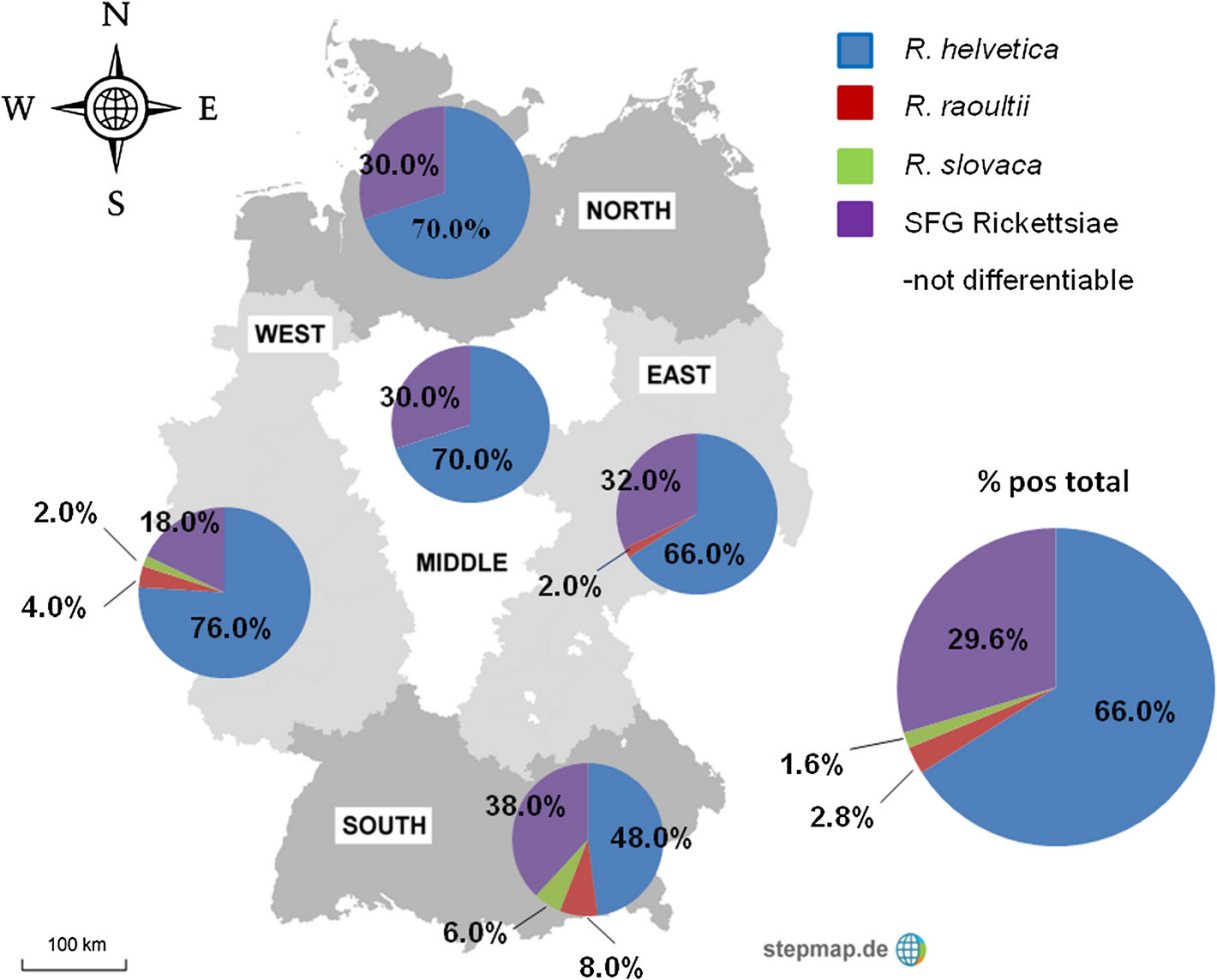
**Division of Germany for sample assessment in five regions and seroprevalence rates of the tested** ***Rickettsia*** **spp. based on the 50 positive samples tested in the five different regions.**

At the screening serum dilution of 1:128, 568 out of 605 (93.9%; CI: 91.7%-95.7%) of the dogs showed antibodies against at least one of the SFG Rickettsiae. Out of the 93.9% anti-*Rickettsia* positive dog specimens 70.4% showed at least twofold higher antibody titres against one *Rickettsia* species and were therefore considered as clearly differentiable. In 23.6% of the dogs equal antibody titers against two and in 5.6% against three rickettsial antigens were observed. Only one specimen (0.4%) was reactive against four of the five *Rickettsia* species (Table 2). All of those were considered as non-differentiable with regard to the antibody-inducing *Rickettsia* species. Considering only the 70.4% clearly differentiable serum samples the total seroprevalence rates were 66.0% (CI: 59.8%-71.9%) for *R. helvetica,* 2.8% (CI: 1.1%-5.7%) for *R. raoultii*, 1.6% (CI: 0.4%-4.1%) for *R. slovaca*, 0.0% (CI: 0.0%-0.1%) for *R. monacensis* and 0.0% (CI: 0.0%-0.1%) for *R. felis.* If all positive serological reactions against the tested rickettsial species, including cross-reactive antibodies, in the randomly selected 250 positive sera (50 from each of the five regions in Germany) were considered, 94.0% (CI: 90.3%-96.6%) for *R. helvetica,* 22.8% (CI: 17.8%-28.5%) for *R. raoultii*, 5.2% (CI: 2.8%-8.7%) for *R. slovaca*, 2.0% (CI: 0.7%-4.6%) for *R. monacensis* and 0.4% (CI: 0.0%-0.2%) for *R. felis* could be observed. Titres varied for *R. helvetica* from 1:128 to 1:4096, for *R. raoultii* from 1:128 to 1:4096, for *R. slovaca* from 1:256 to 1:2048, for *R. monacensis* from 1:256 to 1:2048 and for *R. felis* from 1:128 to 1:512 (Table 3). No statistically significant different infection rates for *R. helvetica* (p = 0.038), *R. slovaca* (p = 0.105) and *R. raoultii* (p = 0.131) were observed comparing the five different regions of Germany (Figure 3). Statistically significant differences for *R. helvetica* were seen comparing north and south (p = 0.042), south and middle (p = 0.042) and south and west (p = 0.007). Comparison of ELISA (Wächter et al., Seroprevalence of Spotted Fever Group Rickettsiae in dogs in Germany, in press) and micro-IFA yielded the following results. All serum samples (n = 605) were tested at a cut-off titre of 1:128. With the ELISA 77.6% (CI: 74.1%-80.9%) of the samples were positive, whereas with the micro-IFA 93.9% (CI: 91.7%-95.7%). Altogether 102 samples were tested positive in the micro-IFA and negative in the ELISA and 6 samples were tested positive in the ELISA and negative in the micro-IFA. Considering this data at a cut-off titre of 1:128 the ELISA shows a sensitivity of 82.0% and a specificity of 83.8%.

**Table 2:** **Seropositivity for SFG Rickettsiae of the dogs with regard to number and association of the different rickettsial species**

**Number of rickettsial species with seropositivity in dogs**	**Rickettsial species**					**No. of positive animals (%)**
	**RH**	**RR**	**RS**	**RM**	**RF**	
**One**	x					165
		x				7
			x			4
				x		0
					x	0
Total						**176 (70.4%)**
**Two**	x	x				32
	x		x			2
	x			x		1
	x				x	20
		x			x	2
		x		x		1
		x	x			1
Total						**59 (23.6%)**
**Three**	x	x	x			4
	x	x		x		2
	x	x			x	7
	x		x		x	1
Total						**14 (5.6%)**
**Four**	x	x	x	x		1
Total						**1 (0.4%)**
					**Total**	**250 (100.0%)**

RH: *Rickettsia helvetica.*

RR: *Rickettsia raoultii.*

RS: *Rickettsia slovaca.*

RM: *Rickettsia monacensis.*

RF: *Rickettsia felis.*

SFG: Spotted fever group*.*

**Table 3:** **Seropositivity for 50 sera/region for** ***R. helvetica*** **,** ***R. raoultii*** **,** ***R. slovaca*** **,** ***R. monacensis and R. felis*** **in serum samples of dogs tested at serial dilutions**

**Rickettsial species**	**RH**		**RR**		**RS**		**RM**		**RF**	
	**No seropositive (%)**		**No seropositive (%)**		**No seropositive (%)**		**No seropositive (%)**		**No seropositive (%)**	
**Titre**	**Differentiable**	**Not differentiable**	**Differentiable**	**Not differentiable**	**Differentiable**	**Not differentiable**	**Differentiable**	**Not differentiable**	**Differentiable**	**Not differentiable**
**1:128**	1 (0.6%)	2 (2.9%)	5 (71.4%)	2 (4.0%)	0 (0.0%)	0 (0.0%)	0 (0.0%)	0 (0.0%)	0 (0.0%)	1 (100.0%)
**1:256**	10 (6.1%)	5 (7.1%)	1 (14.3%)	3 (6.0%)	0 (0.0%)	1 (11.1%)	0 (0.0%)	1 (20.0%)	0 (0.0%)	0 (0.0%)
**1:512**	36 (21.8%)	12 (17.1%)	0 (0.0%)	9 (18.0%)	2 (50.0%)	2 (22.2%)	0 (0.0%)	1 (20.0%)	0 (0.0%)	0 (0.0%)
**1:1024**	86 (52.1%)	29 (41.4%)	0 (0.0%)	23 (46.0%)	1 (25.0%)	5 (55.5%)	0 (0.0%)	1 (20.0%)	0 (0.0%)	0 (0.0%)
**1:2048**	12 (7.3%)	9 (12.9%)	1 (14.3%)	6 (12.0%)	1 (25.0%)	1 (11.1%)	0 (0.0%)	2 (40.0%)	0 (0.0%)	0 (0.0%)
**1:4096**	20 (12.1%)	13 (18.6%)	0 (0.0%)	7 (14.0%)	0 (0.0%)	0 (0.0%)	0 (0.0%)	0 (0.0%)	0 (0.0%)	0 (0.0%)
**No total**	**165**	**70**	**7**	**50**	**4**	**9**	**0**	**5**	**0**	**1**

RH: *Rickettsia helvetica.*

RR: *Rickettsia raoultii.*

RS: *Rickettsia slovaca.*

RM: *Rickettsia monacensis.*

RF: *Rickettsia felis.*

## Discussion

Our study presents the first findings about serological differentiation of rickettsial species in dogs in Germany. The serological differentiation of antibodies against SFG Rickettsiae is difficult due to their strong cross reactivity [12]. The micro-IFA method has already been used by several authors to differentiate between antibodies against SFG-Rickettsiae. Breitschwerdt et al. [44] used micro-IFA for a seroepidemiological study in dogs testing for *R. rickettsii*, *R. rhipicephali, R. montana* and *R. bellii*. In Germany, nine different rickettsial antigens were investigated in hunters to differentiate antibodies against indigenous German SFG Rickettsiae as well as species endemic in regions were the hunters had travelled before [30]. The use of a micro-IFA method enables to a certain extent the differentiation of the antibodies leading to the most dominant serological response. However, a prerequisite is the knowledge of which Rickettsiae occur in the investigated region [45]. We included antigens of SFG Rickettsiae that have been repeatedly detected in Germany and have been described to cause clinical manifestations. *R. massiliae* was not used because a single detection exclusively by molecular tools in Germany was described so far (Table 1) [7]. In this study we examined dogs as potential hosts for the *Rickettsia* species previously described in Germany and showed that, to a certain extent, it is serologically possible to distinguish between them. In 29.6% of the cases the detected antibodies were not differentiable and thus did not allow a clear species allocation. Some authors state that if two or more rickettsial species show distinct fluorescence at the end-titre they may be co-infectious rickettsial species [44,46]. As dogs are more frequently exposed and infested with ticks and the pathogens they harbor than humans, dogs might likely be infected with more than one *Rickettsia* species sequentially during their lifetime, thus explaining the high percentage of not distinguishable seropositive animals (Wächter et al., Seroprevalence of Spotted Fever Group Rickettsiae in dogs in Germany, in press). For the major part (66%) of the samples at least twofold higher antibody titres against *R. helvetica* could be observed. These findings are consistent with the fact that the vector for *R. helvetica* is *Ixodes ricinus*, the most abundant tick species in Germany. Prevalence rates of *R. helvetica* in *I. ricinus* ticks were found in Bavaria, Baden-Wurttemberg, Berlin, Saarland, Lower Saxony and Saxony ranging from 3.5% up to 52.5% (Table 1). The lowest prevalence rates in ticks were found in southern Germany. Accordingly, the lowest seroprevalence rate was found in dogs originating from there. The highest prevalence rates were found in Lower Saxony, which is partly identical with the middle and northern region of Germany where high seroprevalence rates in dogs were found [7,38], (Table 3). Regarding the distribution of dogs with antibodies against *R. raoultii* and *R. slovaca* the distribution areas of the vectors have to be considered. So far, in Germany *R. raoultii* has only been detected in *Dermacentor reticulatus*, *R. slovaca* only in *D. marginatus* [28,39]. Spitalska et al. [47] described in the Slovak Republic that *D. marginatus* and *D. reticulatus* could also be host for *R. raoultii* and *R. slovaca*, respectively. However, comparing the geographic distribution in Germany for *D. reticulatus* and *D. marginatus* [48] with the distribution of seropositive dogs there is a partial concordance. The reason why the seroprevalence comparing the different regions is not statistically significant may be due to the fact that the number of cases were very small (Table 3). *R. monacensis* was detected in low prevalences in ticks (0.2%-0.6%) nearly exclusively in Bavaria, only one study reported *R. monacensis* in ticks in Lower Saxony (Table 1). Regarding the dogs with cross-reactive antibodies against *R. monacensis*, these five dogs were nearly all located in the south of Germany. When comparing the vectors and their infection rates of *I. ricinus* for *R. helvetica* (3.5%-52.5%) and of the cat flea *Ctenocephalides felis* for *R. felis* (25.0%-56.0%) it may be surprising that the seroprevalence rate in dogs for *R. helvetica* is 66.0% and for *R. felis* is 0.0%. However, looking at the infestation of dogs with their vectors, a study about population dynamics of fleas in Germany showed that just 5.1% of the dogs are infested with fleas [49,50]. We showed in our previous study that 91.1% of the examined dogs were infested with ticks up to five ticks/month, and 17.5% even up to 20 ticks/month (Wächter et al., Seroprevalence of Spotted Fever Group Rickettsiae in dogs in Germany, in press). Therefore infestation rates with ticks seem higher than with fleas which may lead to higher seroprevalence rates of *R. helvetica* compared to *R. felis* although the infection rates in the vector population are approximately even (Table 1), [50]. There were no dogs with antibodies exclusively against *R. felis*. The cross-reaction of *R. felis* with other members of the SFG is discussed controversially in the literature. The antibodies have been reported to show stronger reactivity to *R. typhi* from the Typhus Group than to members of the Spotted Fever Group [22,51-53]. Fang and Raoult [54] described that mouse polyclonal antisera against *R. felis* only cross-reacted to members of the SFG Rickettsiae but not to the TG. However, in contrast to the study of Fang and Raoult [54], serum of a classical human case of murine typhus showed exclusive reactivity with TG-Rickettsiae and *R. felis* antigen but none with SFG-Rickettsiae. The same results were obtained when testing a commercially available positive anti-TG Rickettsia positive control (Fuller Laboratories, Fullerton, California, USA) (Wölfel et al., personal communication). In summary this indicates that *R. felis* might be the only SFG Rickettsia to show antigenic relation to the TG Rickettsiae, but can show reactivity to other SFG Rickettsia as well [55]. *R. felis* was the only antigen for which titres above 1:512 were not observed. However, it remains unclear, if the reactivity against several rickettsial antigens including *R. felis* was caused by multiple infections or by cross reaction. The serologic test systems micro-IFA and ELISA are used for seroepidemiologic surveys as well as diagnosing acute cases. The micro-IFA is the gold standard in human medicine, its sensitivity is indicated with over 97%, its specificity with over 99% if a dilution of 1:64 or higher is used [7]. Clements et al. [56] compared the two test systems for diagnosing Rocky Mountain Spotted Fever in humans and in this study the ELISA was as sensitive and as specific as the IFA. Compared to the adapted micro-IFA test in this study the ELISA of the previous study had a moderate sensitivity (82.0%) and specificity (83.8%). These results are in line with non-published results of a comparable study with human sera. In that study, titres lower than 1:128 in an IFA could not be detected by ELISA (Dobler et al., personal communication). One reason for this reduced sensitivity might be that ELISA systems usually are designed for the clinical diagnosis of acute infections with high antibody titres instead of epidemiological studies (Dobler et al., personal communication). Lower antibody titres as expected after a longer post infection interval therefore might remain undetected by ELISA. The sensitivity of a serological assay also depends on the used antigen. In this case the ELISA used in the previous study was produced with *R. rickettsii* antigen, the causative agent of RMSF. Lower sensitivity and specificity rates for antibodies against other rickettsial species might be explained by that, as this *Rickettsia* species is not endemic in Germany. If no differentiation between rickettsial species is needed, ELISA assays might be reliable diagnostic tools for diagnosing acute cases where high antibody titres in sera can be expected and no differentiation between rickettsial species is needed. It is also a good screening tool for high numbers of samples, when the reduction of sensitivity is acceptable. The resulting seroprevalence rate then adds up all the rickettsial species occurring in the tested area. The micro-IFA on the other hand can be used to differentiate several rickettsial species and to determine the different antibody-titres, needed for example to monitor clinical cases.

## Conclusion

This study reports the first results of a reliable serological test differentiating antibodies against rickettsial SFG species in dogs in Germany. The micro-IFA is a suitable tool for dogs to differentiate antibodies of the SFG Rickettsiae. Dogs are able to produce antibodies against SFG-Rickettsiae. The results reflect the natural exposure of dogs in Germany and could be related to the known infection rates in ticks. When compared to the micro-IFA the ELISA of the previous study showed acceptable sensitivity and specificity making both tests suitable for diagnostic purposes. For epidemiological studies the IFA should be preferred due to its higher sensitivity and specificity.
